# Factors Influencing Weight Loss in Young and Middle‐Aged Chinese Patients With Type 2 Diabetes and Overweight/Obesity: A Prospective Cohort Study

**DOI:** 10.1155/nrp/1969758

**Published:** 2026-05-04

**Authors:** Zirui Zheng, Yaxin Li, Tingting Gao, Hui Feng, Qing Jiang, Fanli Zeng, Meie Niu, Lijun Liu, Yanxia Han

**Affiliations:** ^1^ First Affiliated Hospital of Soochow University, Suzhou, China, suda.edu.cn; ^2^ School of Nursing, Soochow University, Suzhou, China, scu.edu.tw

**Keywords:** obesity, overweight, T2DM, weight loss, young adult

## Abstract

**Background:**

The prevalence of young and middle‐aged patients with Type 2 diabetes mellitus (T2DM) and overweight/obesity is increasing in China, yet determinants of weight loss success in this population remain unclear.

**Aims:**

To evaluate weight loss outcomes and identify factors associated with weight loss among young and middle‐aged patients with T2DM and overweight/obesity.

**Methods:**

In this prospective cohort study, 282 patients with T2DM and overweight/obesity were recruited between March and September 2024. Baseline demographic, anthropometric, and psychosocial data were collected before discharge. Body weight was reassessed 3 months after discharge, and percentage weight loss was calculated. Participants were categorized into success (≥ 5% weight loss) and failure (< 5% weight loss) groups. Factors associated with weight loss were identified using univariate analysis, random forest importance ranking, LASSO regression, and multivariable logistic regression.

**Results:**

Using ≥ 5% weight loss as the clinical target, the overall success rate was 43.7%, with gender‐specific rates of 39.5% in males and 58.0% in females. Random forest and LASSO analyses identified six key predictors: diabetes duration, extraversion, agreeableness, conscientiousness, diabetes self‐efficacy, and social support. Multivariable logistic regression showed that diabetes duration (OR = 6.511, 95% CI: 1.92–22.05), extraversion (OR = 0.847, 95% CI: 0.73–0.98), agreeableness (OR = 1.228, 95% CI: 1.05–1.43), conscientiousness (OR = 1.254, 95% CI: 1.09–1.44), and diabetes self‐efficacy (OR = 1.062, 95% CI: 0.993–1.14) were significant predictors of weight loss success.

**Conclusions:**

Weight loss outcomes among young and middle‐aged patients with T2DM and overweight/obesity were suboptimal and differed by gender. Diabetes duration, personality traits, and diabetes self‐efficacy were independent predictors of weight loss success.

## 1. Introduction

According to the 10th edition of the International Diabetes Federation Diabetes Atlas [1], the global prevalence of diabetes has reached 537 million individuals and is projected to rise to 783 million by 2045. In China, the number of individuals with diabetes has surged to 140 million, with a prevalence rate of 11.2% [2]. More concerning, the disease burden of Type 2 diabetes mellitus (T2DM) among young and middle‐aged populations in China has been rising rapidly, particularly in those with overweight/obesity [3]. Rapid socioeconomic development has driven profound lifestyle changes in this demographic, including imbalanced diets, reduced physical activity, increased consumption of high‐calorie foods, smoking, alcohol use, sedentary behavior, and irregular sleep patterns, all contributing to a growing burden of T2DM and overweight/obesity [4]. In addition, China has observed an alarming youth‐oriented trend in disability‐adjusted life years (DALYs) and mortality attributable to high BMI‐related T2DM, with this disease burden projected to continue escalating [5]. These findings underscore the strong association between overweight/obesity and T2DM.

Weight loss, defined as the intentional and scientifically guided reduction of body mass to improve overall health and mitigate risks associated with overweight/obesity, plays a pivotal role in diabetes management [6]. Current international guidelines emphasize the importance of weight reduction, particularly among young and middle‐aged patients with T2DM. For example, most guidelines recommend that a 5% weight loss can improve glycated hemoglobin and blood pressure levels; a 5%–10% loss yields further metabolic benefits; and sustained weight loss of more than 10% can induce T2DM remission, improve cardiovascular outcomes, and reduce mortality [7, 8]. A dose‐dependent relationship between weight loss and diabetes reversal has been demonstrated, with complete diabetes remission rates reaching 79.1% among individuals achieving more than 30% weight loss and 49.6% among those losing 20%–29% of their body weight, and each 1% reduction in body weight is associated with a 2.17% increase in the likelihood of achieving complete diabetes remission [9].

Weight loss strategies for patients with T2DM and overweight/obesity primarily include inpatient education, lifestyle interventions, pharmacotherapy, and bariatric surgery [10]. Despite their varying degrees of clinical efficacy, real‐world data highlight several challenges, such as suboptimal treatment adherence, inadequate self‐management capacity, and significant inter‐individual variability in therapeutic responses [11–13], all of which contribute to unsatisfactory weight loss outcomes, including suboptimal weight loss and weight regain.

Previous literature has identified multiple factors influencing weight loss outcomes in patients with T2DM, including individual factors (e.g., educational attainment, self‐efficacy, personality traits, and occupational status) [13, 14]; socio‐environmental factors (e.g., social support, occupational demands and stressors, and socioeconomic status) [15–17]; and disease‐related factors (e.g., diabetes duration, treatment modalities, and comorbidities) [18, 19]. However, most existing studies have employed a cross‐sectional design, limiting the ability to assess causal relationships. Moreover, the impact of inpatient education on weight loss remains poorly understood, particularly among young and middle‐aged Chinese patients with T2DM and overweight/obesity.

To address these gaps, this prospective cohort study aims to identify factors influencing weight loss and to evaluate the effectiveness of inpatient education in improving target weight loss achievement in this population. The findings are expected to provide valuable evidence to inform and optimize individualized weight management strategies, thereby helping to reduce the long‐term burden of diabetes in clinical practice.

## 2. Methods

### 2.1. Study Design

This study was a prospective cohort study conducted from March to September 2024 through convenience sampling at four tertiary general hospitals in Suzhou, Jiangsu Province, eastern China.

### 2.2. Participants

A total of 282 young and middle‐aged patients with T2DM and overweight/obesity were enrolled from the four hospitals described above. All participants were hospitalized during the study period for glycemic control optimization or complication screening. The inclusion criteria for the study were as follows: (1) Diagnosis of T2DM based on the 1999 World Health Organization diagnostic criteria: presence of classic symptoms (polyphagia, polydipsia, polyuria, or unexplained weight loss) and venous plasma glucose meeting at least one of the following: fasting plasma glucose ≥ 7.0 mmol/L; random plasma glucose ≥ 11.1 mmol/L; 2‐h oral glucose tolerance test glucose ≥ 11.1 mmol/L. (2) Diagnosis of overweight/obesity according to the Guidelines for Prevention and Control of Overweight and Obesity in Chinese Adults [20]: overweight: BMI ≥ 24 kg/m^2^ to < 28 kg/m^2^, obesity: BMI ≥ 28 kg/m^2^. (3) Age 18–59 years (inclusive), aligning with the United Nations definition of young and middle‐aged adults. (4) Patients who received a standardized face‐to‐face weight loss education session from a certified diabetes specialist nurse prior to discharge, covering diet, physical activity, behavioral strategies, and medication adherence. Exclusion criteria were (1) comorbid severe hepatic/renal dysfunction or malignancy, (2) history of bariatric surgery, (3) pregnancy or lactation, and (4) severe cognitive impairment or intellectual/hearing disabilities.

In this study, we explored factors influencing weight loss control among young and middle‐aged patients with T2DM and overweight/obesity within 3 months post‐discharge. Before discharge, demographic and clinic data were collected, body weight was measured using a calibrated weight scale (RGZ‐120; Suhong Medical Instrument Co., Jiangsu, China), and psychological/social assessments were conducted using standardized instruments. At 3 months post‐discharge, patients were reminded by telephone to attend the outpatient clinic within 1 week, where body weight was re‐measured using the same calibrated weight scale model. The weight loss percentage was calculated based on body weight before discharge and weight 3 months post‐discharge (weight loss percentage = [initial weight − weight 3 months post‐discharge]/initial weight × 100%). Participants were categorized into two groups: the weight loss failure group (< 5% weight loss) and weight loss success group (≥ 5% weight loss).

### 2.3. Measurements

#### 2.3.1. General Information

Demographic and clinical data were collected via questionnaires, including age, gender, health care payment, educational level, marital status, occupation, household income, comorbidities, family history of diabetes, treatment modalities, and diabetes duration.

#### 2.3.2. Chinese Version of the Ten‐Item Personality Inventory (TIPI‐C)

We used the TIPI‐C revised by Chinese scholar Li [21] in this study. This 10‐item scale assesses five personality dimensions: openness, extraversion, conscientiousness, agreeableness, and neuroticism. Items are rated on a 7‐point Likert scale, with higher scores indicating stronger traits. The internal consistency coefficients for the five dimensions ranged from 0.600 to 0.670, values generally regarded as acceptable in measurement. The test–retest reliability ranged from 0.410 to 0.770, suggesting that the reliability of the measures falls within the moderate to good range.

#### 2.3.3. Self‐Efficacy for Diabetes Scale (SED)

The original SED was developed by Lorig et al. at Stanford University [22], and the Chinese version was adapted by Sun et al. [23]. The 8‐item scale evaluates self‐efficacy in diet management, exercise, and glucose control using a 10‐point Likert scale. Higher average scores reflect better self‐efficacy. The Chinese version demonstrated a Cronbach’s α of 0.828.

#### 2.3.4. Perceived Social Support Scale (PSSS)

The PSSS was developed by Zimet et al. [24], and the Chinese version was validated by Huang et al. [25]. This 12‐item scale comprises three domains, including family support, support from friends, and support from others, scored on a 7‐point Likert scale (total score: 12–84). Scores ≤ 36 indicate low social support, 37–60 moderate social support, and 61–84 high social support. The Chinese version showed a Cronbach’s α of 0.880.

#### 2.3.5. Work Stressors Scale

The 21‐item scale developed by Li [26] assesses occupational stress across five domains, concluding work tasks, working environment, interpersonal relationships, management affairs, and employee qualities. Items are rated on a 5‐point Likert scale, with higher scores reflecting greater stress. The scale demonstrated a Cronbach’s α of 0.780.

### 2.4. Ethical Considerations

This prospective cohort study was approved by the Ethics Committee of the First Affiliated Hospital of Soochow University (approval no. 306‐2024). Written informed consent was obtained from all participants. This study was conducted in accordance with the Declaration of Helsinki.

### 2.5. Data Collection

Two well‐trained investigators surveyed the hospital setting. Patients meeting the inclusion criteria were screened and informed about the study objectives and procedures. After obtaining informed consent, body weight, demographic information, clinical data, and scale‐based data were recorded before discharge. At the 3‐month follow‐up, patients received telephone reminders to return to the hospital for weight reassessment. To minimize inter‐device variability, we purchased and distributed the same weighing scale (RGZ‐120) to four centers. These scales were calibrated daily using standard weights prior to each measurement session to ensure accuracy and consistency across sites and time points. All questionnaires were completed face‐to‐face, with uniform instructions. For patients unable to self‐complete the questionnaires, investigators administered survey items verbally, without leading or suggestive language. Completed questionnaires were immediately reviewed for completeness; missing items were clarified and supplemented on‐site.

### 2.6. Statistical Methods

Statistical analysis was conducted using IBM SPSS 27.0 and R Studio (Version 4.5.1) for data organization and analysis. Quantitative data that followed a normal distribution are expressed as mean ± standard deviation, and data not conforming to a normal distribution are reported as median and interquartile range. Qualitative data are presented as frequency and percentage. The outcome variable was the percentage of weight loss after 3 months post‐discharge, coded as “weight loss failure group = 1” and “weight loss success group = 2.” Univariate analysis was performed using the chi‐square test or Mann–Whitney *U* test. The variance inflation factor (VIF) was used to check for multicollinearity among variables.

Random forest (RF), an ensemble learning method based on decision trees, offers robustness against overfitting, interpretability, and the ability to handle high‐dimensional data while assessing feature importance. In this study, we implemented RF in R Studio, incorporating 13 variables with statistically significant differences in univariate analysis to rank their importance. Cross‐validation and a grid search were used to optimize critical parameters. The data were split into a development set (80%) and a test set (20%). The model was trained on the development set and validated on the test set. Variable significance was evaluated using the mean decrease in the Gini coefficient (MDG), where a higher value indicates greater variable importance. The model’s performance was assessed using the receiver operating characteristic (ROC) curve and confusion matrix to aid in further optimization.

Least absolute shrinkage and selection operator, also known as LASSO regression, is a shrinkage estimation method. LASSO constructs a penalty function to shrink the coefficients of unimportant variables to zero, facilitating variable selection. In LASSO regression, the *λ* value from cross‐validation determines which variables to retain. One can choose lambda.min, which minimizes the average error, or lambda.1se, which gives the most regularized model where the error is within one standard error of the minimum. In this study, LASSO regression was used for variable shrinkage, opting for lambda.1se as it provides a more parsimonious model, helping to avoid overfitting. Finally, the top six selected variables were included in binary logistic regression for multivariate analysis. Statistical significance was set at *p* < 0.05.

## 3. Results

### 3.1. Participant Inclusion

**FIGURE 1:**
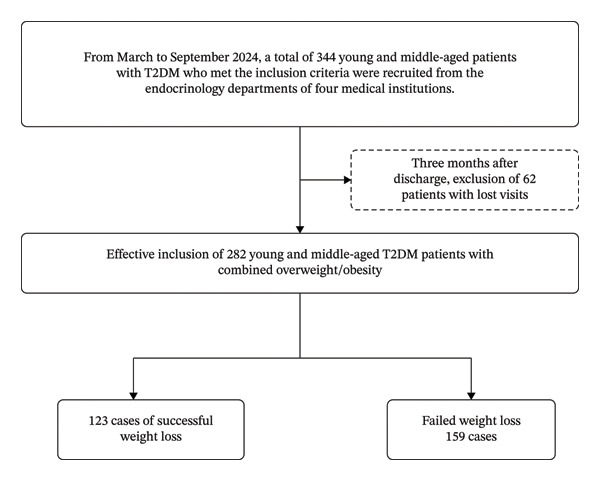
Flowchart for inclusion of study participants.

This study included 344 eligible young and middle‐aged patients with T2DM who visited the endocrinology departments of four medical institutions in Suzhou, Jiangsu Province of China from March to September 2024. Among them, 62 patients were lost to 3‐month follow‐up or did not return for subsequent visits, resulting in a loss to follow‐up rate of 18%. The effective data included in the analysis comprised 282 patients, with an effective data rate of 82%. A flowchart illustrating the inclusion of study participants is presented in Figure 1.

### 3.2. Univariate Analysis of Participants’ Weight Loss

Among the included 282 young and middle‐aged patients with T2DM and comorbid overweight/obesity, 220 were men (78%) and 62 were women (22%). The mean patient age was 40.3 ± 9.6 years, and the average weight loss percentage was 3.46 ± 3.44%. Of the total, 123 patients (43.6%) successfully lost weight (weight loss ≥ 5%) and 159 patients (56.4%) failed to successfully lose weight (weight loss < 5%).

Univariate analysis identified 13 variables associated with weight loss, including gender, health care payment, education level, occupational status, diabetes treatment modality, diabetes duration, extraversion personality trait, agreeableness personality trait, conscientiousness personality trait, openness personality trait, diabetes self‐efficacy, social support, and work stressors (*p* < 0.05) (Table 1). A multiple collinearity diagnosis was performed using the 13 statistically significant independent variables in univariate analysis. The results showed that the VIFs of all independent variables were < 5, and the tolerance values were > 0.2, indicating no collinearity among the independent variables.

**TABLE 1:** Baseline demographic characteristics of participants and univariate analysis of weight loss control (*n* = 282).

Variables	Weight loss failure group (*n* = 159)	Weight loss success group (*n* = 123)	*Z/* *χ* ^2^	*p*
Age (*M* ± SD)	40.8 ± 9.4	39.6 ± 9.9	−0.907	0.364
Gender			6.745	**0.009**
Male, *n* (%)	133 (83.6)	87 (70.7)		
Female, *n* (%)	26 (16.4)	36 (29.3)		
Health care payment			12.990	**< 0.001**
Medical insurance, *n* (%)	120 (75.5)	113 (91.9)		
Self‐payment, *n* (%)	39 (24.5)	10 (10)		
Education level			−2.378	**0.017**
Primary school and below, *n* (%)	10 (6.3)	8 (6.5)		
Middle school and secondary specialized school, *n* (%)	47 (29.6)	31 (25.2)		
High school and college, *n* (%)	69 (43.4)	34 (27.6)		
Bachelor’s degree and above, *n* (%)	33 (20.8)	50 (40.7)		
Marital status			0.706	0.703
Unmarried, *n* (%)	18 (11.3)	15 (12.2)		
Married, *n* (%)	131 (82.4)	103 (83.7)		
Divorced, *n* (%)	10 (6.3)	5 (4.1)		
Living situation			1.411	0.494
Living alone, *n* (%)	25 (15.7)	17 (13.8)		
Living with spouse, *n* (%)	130 (81.8)	105 (85.4)		
Living with parents/children, *n* (%)	4 (2.5)	1 (0.8)		
Occupational status			13.654	**0.001**
Employed, *n* (%)	113 (71.1)	63 (51.2)		
Freelance, *n* (%)	39 (24.5)	44 (35.8)		
Unemployed, *n* (%)	7 (4.4)	16 (13.0)		
Monthly household income per capita			−1.409	0.159
Less than 1000 RMB, *n* (%)	7 (4.4)	4 (3.3)		
1000–3000 RMB, *n* (%)	22 (13.8)	10 (8.1)		
3000–5000 RMB, *n* (%)	39 (24.5)	30 (24.4)		
More than 5000 RMB, *n* (%)	91 (57.2)	79 (64.2)		
Cardiovascular and cerebrovascular diseases			1.115	0.282
Yes, *n* (%)	98 (61.6)	68 (55.3)		
No, *n* (%)	61 (38.4)	55 (44.7)		
Family member with diabetes			0.260	0.610
Yes, *n* (%)	94 (59.1)	69 (56.1)		
No, *n* (%)	65 (40.9)	54 (43.9)		
Diabetes treatment			25.407	**< 0.001**
Oral medication only, *n* (%)	70 (44.0)	90 (73.2)		
Insulin only, *n* (%)	17 (10.7)	10 (8.1)		
Combination therapy, *n* (%)	72 (45.3)	23 (18.7)		
Diabetes duration			73.187	**< 0.001**
Less than 5 years, *n* (%)	52 (32.7)	103 (83.7)		
5–10 years, *n* (%)	67 (42.1)	14 (11.4)		
More than 10 years, *n* (%)	40 (25.2)	6 (4.9)		
TIPI‐C, median (IQR)				
Extraversion, median (IQR)	10 (8–12)	8 (6–9)	−7.121	**< 0.001**
Agreeableness, median (IQR)	5 (4–8)	8 (7–10)	−7.390	**< 0.001**
Conscientiousness, median (IQR)	8 (6–10)	11 (10–12)	−8.767	**< 0.001**
Neuroticism, median (IQR)	8 (7–9)	8 (7–9)	−0.062	0.950
Openness to experiences, median (IQR)	8 (8–11)	12 (9–13)	−7.287	**< 0.001**
SED, median (IQR)	28 (18–37)	45 (36–52)	−9.548	**< 0.001**
PSSS, median (IQR)	54 (45–65)	70 (56–79)	−6.377	**< 0.001**
Work Stressor Scale, median (IQR)	69 (45–84)	58 (31–78)	−2.576	**0.010**

*Note:* The bold values indicate statistical significance (*p* < 0.05) in the univariate analysis.

### 3.3. Factors Influencing Weight Loss Among Participants

#### 3.3.1. Variable Importance Ranking

**FIGURE 2:**
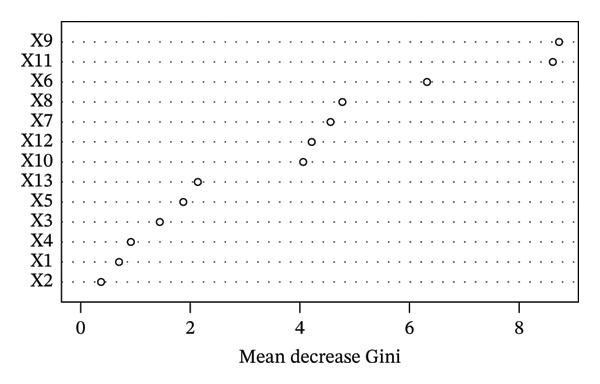
Single‐factor importance ranking in random forest analysis. The order of importance from highest to lowest is: conscientiousness, diabetes self‐efficacy, diabetes duration, agreeableness, extraversion, social support, openness, work stressors, diabetes treatment modalities, education level, occupational status, gender, and health care payment.

**FIGURE 3:**
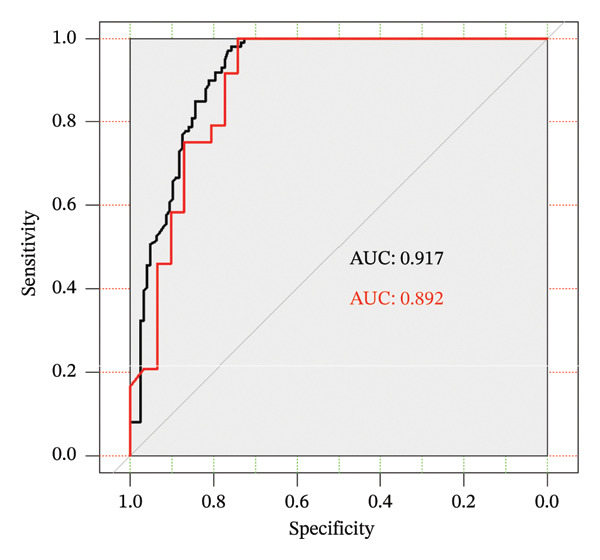
ROC curves for the training set and test set.

Using weight loss percentage as the dependent variable and statistically significant variables from univariate analysis as independent variables, an RF model was constructed with the “RandomForest” package in R Studio. Feature importance was evaluated using the MDG, which quantifies the reduction in Gini impurity during node splitting in decision trees. Higher MDG values indicate greater variable importance. Variable coding and assignments are detailed in Table 2. The results showed that in order of importance from highest to lowest, the variables were conscientiousness, diabetes self‐efficacy, diabetes duration, agreeableness, extraversion, social support, openness, work stressors, diabetes treatment modalities, education level, occupational status, gender, and health care payment (Figure 2). ROC curves generated using the “pROC” package in R demonstrated area under the ROC curve (AUC) values of 0.917 (training set) and 0.892 (test set) (Figure 3). Model performance evaluated via a confusion matrix showed satisfactory sensitivity, specificity, and accuracy (Table 3).

**TABLE 2:** Variable coding and assignment in the random forest model.

Independent variable	Coding	Assignment	Mean decrease in Gini
Gender	X_1_	Male = 1, Female = 2	0.702
Health care payment method	X_2_	Medical insurance = 1, Self‐payment = 2	0.375
Education level	X_3_	Primary school or below = 1; Junior high school/Secondary specialized school = 2; High school/College = 3; Bachelor’s degree and above = 4.	1.443
Occupational status	X_4_	Employed = 1, Freelance = 2, Unemployed = 3	0.920
Diabetes treatment method	X_5_	Oral medication only = 1, Insulin only = 2, Combination therapy = 3	1.871
Diabetes duration	X_6_	Less than 5 years = 1, Less than 10 years = 2, More than 10 years = 3	6.318
Extraversion	X_7_	Raw score input	4.564
Agreeableness	X_8_	Raw score input	4.776
Conscientiousness	X_9_	Raw score input	8.729
Openness to experiences	X_10_	Raw score input	4.056
SED	X_11_	Raw score input	8.621
PSSS	X_12_	Raw score input	4.217
Workplace stressor scale	X_13_	Raw score input	2.121
Weight loss control	Y	Weight loss failure = 1, Weight loss success = 2	—

**TABLE 3:** Comparison of model performance in the training set and test set.

	Training set	Test set
AUC	0.91	0.89
Sensitivity	1.00	1.00
Specificity	0.71	0.74
Accuracy	0.83	0.85
Kappa	0.68	0.71

#### 3.3.2. Variable Selection

**FIGURE 4:**
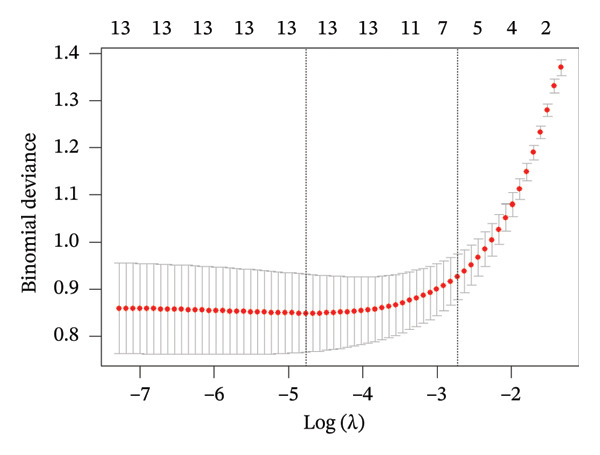
Characteristic variable screening for LASSO regression. The left vertical dashed line in the figure represents lambda.min; the right vertical dashed line corresponds to lambda.1se. The model exhibited minimal deviation fluctuation within the interval between lambda.1se and lambda.min.

Based on the variable importance ranking, LASSO regression analysis was performed on the 13 statistically significant variables identified in univariate analysis using the “glmnet” package in R Studio (Figure 4). When the lambda (*λ*) value is 0.0658, the error is minimized, corresponding to six influencing factors. Consequently, the top six variables—diabetes duration, extraversion, agreeableness, conscientiousness, diabetes self‐efficacy, and social support—were included in subsequent binary logistic regression analysis.

#### 3.3.3. Multivariate Analysis of Factors Influencing Participants’ Weight Loss

Binary logistic regression analysis was conducted, with weight loss percentage as the dependent variable and the six highest‐ranked variables from the RF model as independent variables. The results demonstrated that diabetes duration, extraversion, agreeableness, conscientiousness, and diabetes self‐efficacy were significant predictors of weight loss in young and middle‐aged patients with T2DM who had comorbid overweight/obesity (Table 4).

**TABLE 4:** Results of binary logistic regression for factors influencing weight loss.

Variables	*β* coefficient	OR	95% CI	*p* value
Diabetes duration^∗^	1.874	6.511	1.92–22.05	0.003
Extraversion	−0.166	0.847	0.73–0.98	0.028
Agreeableness	0.206	1.228	1.05–1.43	0.008
Conscientiousness	0.226	1.254	1.09–1.44	0.002
SED	0.060	1.062	0.993–1.04	< 0.001

^∗^Diabetes duration < 5 years was the reference category.

### 3.4. Gender Differences in Baseline Psychological Characteristics

To explore potential explanations for the observed gender disparity in weight loss success, we compared baseline psychological characteristics between male and female using Mann–Whitney *U* tests (Table 5). Female patients had significantly higher scores in agreeableness (*p* = 0.019). No significant gender differences were observed for extraversion, conscientiousness, diabetes self‐efficacy, and social support.

**TABLE 5:** Baseline psychological characteristics by gender.

Variables	Male (*n* = 220)	Female (*n* = 62)	*Z*	*p* value
Extraversion	8.00 (7.00–11.00)	8.50 (8.00–10.00)	−0.354	0.723
Agreeableness	6.00 (4.00–9.00)	7.00 (5.75–9.00)	−2.351	**0.019**
Conscientiousness	9.00 (7.00–11.00)	10.50 (8.00–12.00)	−1.955	0.051
SED	35.00 (25.00–46.00)	34.00 (27.75–44.25)	−0.278	0.781
PSSS	59.00 (46.00–74.00)	59.00 (52.00–75.00)	−0.849	0.396

*Note:* The bold values indicate statistical significance (*p* < 0.05).

## 4. Discussion

The findings of this study showed that with a 5% weight loss within 3 months post‐discharge set as the weight loss goal, the overall successful weight loss attainment rate among our participants was 43.7%, with a mean weight loss of 3.46%. The average weight loss percentage in the DiRECT study in the United Kingdom was 12.5% [27], which differs somewhat from the results of our study. This divergence may stem from methodological differences. For example, the DiRECT trial implemented intensive interventions including very low‐calorie diets and long‐term professional weight control guidance, supported by robust follow‐up monitoring and high patient adherence [27]. The present study followed standard discharge instructions commonly implemented in clinical practice, incorporating an additional educational manual as supporting information. Previous studies [28–30] have confirmed that health education alone has limited efficacy in driving behavioral changes and that sustained improvement requires integrated strategies. It is also worth noting that the weight loss attainment rates for male and female patients were 39.5% and 58%, reflecting an 18.5% gender difference. This gender heterogeneity may be related to sociocultural factors; young and middle‐aged women with T2DM pay greater attention to their body image and body weight management, which are related to social identity [31, 32]. These female patients may also be more willing to engage in healthy behaviors, such as dietary recording and regular exercise in their daily lives [33]. Additionally, our study revealed that female patients had significantly higher scores in agreeableness, a personality trait characterized by cooperation, trust, and compliance. This psychological characteristic may further facilitate adherence to dietary and behavioral recommendations, partially explaining the observed gender disparity in weight loss outcomes [34]. Based on the above findings, implementing personalized and specific weight control strategies according to gender is recommended. Strengthening positive incentive mechanisms for female patients with T2DM and overweight/obesity and focusing on health cognition remodeling for male patients is important.

This study systematically revealed factors influencing weight loss control in young and middle‐aged patients with T2DM and overweight/obesity by integrating machine learning with traditional regression models. Methodologically, the dual variable selection strategy, combining RF (based on the MDG) and LASSO regression (optimal *λ* = 0.065 with minimal cross‐validation error), demonstrated distinct advantages [35, 36]. The former identified nonlinear associations through feature importance ranking, and the latter eliminated multicollinearity interference via L1 regularization. Together, these methods were used to verify the stability of six core variables: conscientiousness personality trait, diabetes self‐efficacy, agreeableness personality trait, diabetes duration, extroversion personality trait, and social support. The final binary logistic regression model further confirmed five independent predictors, suggesting that disease characteristics (duration), psychological personality dimensions (conscientiousness, agreeableness, and extroversion), and disease management ability (self‐efficacy) constitute a synergistic network influencing weight loss effects.

In this study, patients with a diabetes duration of 5–10 years had a significantly higher likelihood of achieving weight loss success compared to those with duration < 5 years (OR = 6.511, 95% CI: 1.92–22.05), suggesting that medium‐term disease duration may influence weight management outcomes. This finding contrasts with previous studies which have reported that shorter diabetes duration is associated with better weight loss and metabolic outcomes [37, 38]. The discrepancy may be explained by cultural and behavioral factors specific to our Chinese cohort. Chen et al. [39] demonstrated that Chinese patients with longer diabetes duration are more adept at translating health knowledge into effective self‐management. In addition, patients who develop healthy habits and maintain strong motivation over years of disease management are better able to sustain self‐care behaviors [40]. Thus, enhanced disease awareness and self‐management routines may explain why patients with 5–10 years of diabetes duration in our cohort achieved greater weight loss success. Clinically, the findings of the present study highlight the need for early metabolic management in newly diagnosed patients and affirm that longer‐duration patients can also benefit from weight loss efforts. Nurses should be supported in providing evidence‐based weight management counseling to both groups.

Our study found that higher extraversion scores were correlated with an increased risk of weight loss failure, whereas elevated agreeableness and conscientiousness scores predicted higher success rates, which is consistent with previous research findings [41–43]. Eysenck’s personality theory suggests that extroverts have a lower level of cortical arousal and seek external stimulation (such as social activities) to increase excitement, which can influence their dietary choices [44]. The study by Buse et al. [45] observed a weak correlation between extraversion and dietary restraint, indicating that although extraverts may be more likely to adhere to dietary rules in social settings, they may also choose high‐calorie foods owing to external social pressure. This suggests that extraversion can be a double‐edged sword in terms of social behavior, reflecting the complexity of behavioral regulation. Additionally, the dopamine system of extroverts is highly sensitive [44], which may enhance their pursuit of reward. Keller et al. [46] confirmed that highly extroverted individuals are often more sensitive to immediate rewards. This aligns with the “distracted attention hypothesis” in social facilitation theory [47], in which extraverts’ susceptibility to external stimuli may impair self‐regulation.

Our findings also suggested that individuals with conscientiousness or agreeableness traits are more likely to successfully achieve weight loss. This discovery is highly consistent with previous research [45]. From a behavioral mechanism perspective, individuals with the conscientiousness trait demonstrate stronger goal persistence and self‐regulatory behaviors, and these individuals are more likely to develop healthy behavioral cognition and adherence [48]. A positive correlation has been found between conscientiousness and regular exercise, blood glucose monitoring, and self‐management behaviors among patients with T2DM [34]. Additionally, agreeableness influences weight loss via social interaction. Patients with high agreeableness possess better social collaboration advantages, and their altruism and compliance make them more willing to cooperate with weight loss control plans and establish long‐lasting patient–doctor alliances [34].

In clinical practice, these findings suggest that incorporating personality assessment into weight loss interventions for young and middle‐aged patients with T2DM may help tailor behavioral strategies. Brief personality assessments could guide nurses in selecting appropriate intervention approaches. For example, individuals with high conscientiousness may benefit from structured goal‐setting and self‐monitoring, whereas those with high agreeableness may respond better to collaborative care and family involvement. Patients with low extraversion may prefer individualized counseling. Personality‐informed strategies are feasible and may enhance patient engagement and adherence in weight management programs.

This study demonstrated that for every 1‐point increase in the total score for diabetes self‐efficacy, the likelihood of successful weight loss increased by 6.2% (OR = 1.062, 95% CI: 0.993–1.04). Although the OR value is close to 1, its high significance suggests a small but stable cumulative effect. The Chinese Health Commission announced the implementation of Year of Weight Management to raise awareness about weight management among the public in 2024–2026 [49]. This initiative is aimed at assisting individuals to gradually improve their self‐efficacy through scientific understanding, behavioral practices, and social interaction. Individuals with high self‐efficacy are more likely to adhere to a long‐term healthy lifestyle [50], avoiding weight fluctuations. Structured educational models such as workshops, peer education with sharing of weight loss experiences, and digital self‐monitoring tools [51–53] can be provided to enhance self‐efficacy, thereby improving weight loss among young and middle‐aged patients with T2DM and overweight/obesity.

Although social support was identified as an important predictor in both RF and LASSO regression analyses, it was not statistically significant in the logistic regression model. This discrepancy may have several explanations. First, social support may influence weight loss indirectly through psychological mediators, particularly diabetes self‐efficacy, which remained significant in the final model. According to social cognitive theory [54], supportive environments can enhance individuals’ confidence in performing health behaviors, thereby facilitating behavioral change. Second, RF models can capture complex nonlinear relationships and interactions that are not specified in conventional logistic regression [55]. Social support may therefore interact with factors such as diabetes duration, masking its independent effect. Future studies using structural equation modeling or prespecified interaction terms are needed to further examine these potential mediation and interaction mechanisms.

### 4.1. Limitations

This study has several limitations. First, the use of convenience sampling restricted recruitment of participants to four hospitals within a single geographic region, limiting the generalizability of the findings. Future studies should include patients from diverse regions and health care settings to explore potential variations in weight management outcomes. Second, although influencing factors were selected based on a literature review, the scope was limited, and important psychological or background factors may have been omitted; therefore, the study results should be interpreted with caution. Future studies should use a robust theoretical framework to guide the investigation and adopt mixed methods to comprehensively explore multiple influencing factors. Third, weight management in patients with T2DM inherently involves dual goals of glycemic control and weight reduction. However, in this study, we primarily focused on weight‐related metrics. Future research should integrate comprehensive metabolic indicators (e.g., blood glucose, lipid profiles, blood pressure) to holistically evaluate intervention efficacy. Fourth, personality traits were assessed using the brief TIPI‐C, which has relatively low internal consistency. Its limited reliability may have attenuated the observed associations. Future studies should consider using more comprehensive personality measures with higher reliability. Finally, the 3‐month follow‐up only reflects short‐term weight loss outcomes, and the long‐term sustainability of these effects remains unclear. Studies with longer follow‐up periods are needed to examine long‐term weight management trajectories.

## 5. Conclusion

This study elucidated the synergistic influence of metabolic characteristics (diabetes duration), psychological personality traits (conscientiousness, agreeableness, and extroversion), and self‐management abilities (diabetes self‐efficacy) on weight loss control among young and middle‐aged patients with T2DM and overweight/obesity. Both RF and LASSO regression confirmed the stability of the aforementioned core variables. Among them, the strong effect of diabetes duration suggests the need to develop metabolic enhancement strategies targeting patients with long disease durations. Moreover, the differentiated roles of conscientious and extroverted personalities highlight the need for precise psychological intervention. Although social support did not reach the significance threshold, its potential mediating effect deserves further exploration. In clinical practice, we recommend integrating stratified management based on disease duration, personalized interventions based on personality traits, and self‐efficacy enhancement training to develop an evidence‐based, multi‐dimensional, individualized weight loss program for young and middle‐aged patients with T2DM and overweight or obesity.

## Author Contributions

Conception and design, acquisition of data, and analysis and interpretation of data: Zirui Zheng, Yaxin Li, and Tingting Gao. Data collection and interpretation: Hui Feng, Qing Jiang, and Fanli Zeng. Interpreting data and writing: Yaxin Li, Tingting Gao, and Meie Niu. Manuscript review and revision: Meie Niu, Lijun Liu, and Yanxia Han.

## Funding

No funding was received for this study.

## Conflicts of Interest

The authors declare no conflicts of interest.

## Data Availability

The data that support the findings of this study are available upon request from the corresponding author. The data are not publicly available due to privacy or ethical restrictions.
